# High order Fano resonance in the time domain for a freezing water microdroplet

**DOI:** 10.1038/s41598-024-74425-1

**Published:** 2024-10-15

**Authors:** Igor V. Minin, Oleg V. Minin, Song Zhou, Boris S. Luk’yanchuk

**Affiliations:** 1 National Research Tomsk Polytechnical University, Tomsk, 634050 Russia; 2 Jiangsu Key Laboratory of Advanced Manufacturing Technology, Faculty of Mechanical and Material Engineering, Huaiyin Institute of Technology, Huai’an, 223003 China; 3 Faculty of Physics, Lomonosov Moscow State University, Moscow, 119991 Russia

**Keywords:** Optical physics, Micro-optics

## Abstract

Fog is a collection of micro drops of water suspended in the air, formed as a result of cooling of moist air. In supercooled air, water droplets freeze, forming ice fog at air temperatures below − 10–15° C. As the ice drop freezes, it forms a core-shell structure. In such a particle, a high-Q Fano resonance is possible, which entails the formation of a magnetic pulse. Our theoretical calculations have predicted the time-dependent formation of Fano resonances in a freezing the outside in water droplet. Time-varying unconventional Fano resonance with magnetic field enhancement yield new method to manipulate light–matter interactions in a freezing water droplet. To the best of our knowledge this mechanism was not discussed previously.

## Introduction

Research on the formation of fog in the liquid-vapor system was twice awarded the Nobel Prize in Physics^1,2^. Another phase transition associated with the transformation of fog into ice plays an important role in atmospheric physics^3^. Laser ablation of deuterium ice crystals is used to generate neutrons^4^. Unusual optical phenomena are possible in freezing fog. The fact is that a small drop of liquid with an ice shell is a resonator in which, under certain conditions, Fano resonances can be excited, leading to the generation of large electric and magnetic fields^5–7^. As a result, at a certain stage of freezing, the drop emits a strong magnetic pulse. Since fog can be thought of as a natural metamaterial with an optical neural network, the exchange of magnetic impulses between droplets can, in principle, lead to a complex system response, such as the fantastic phenomena described in the famous novel “The Black Cloud” by Fred Hoyle^8^. Experimental studies^9^and the literature cited there show that the phase transition in small droplets of supercooled water is an extremely complex and rapid multi-stage process. However, the theoretical model^9^ does not include effects associated with magnetic fields formed in a freezing drop. In this regard, it seems relevant to us to theoretically evaluate the effects of the magnetic response in a freezing drop of fog.

## Model

The problem of freezing spherical drops with a large Bond number, which characterizes the action of gravity and surface tension in a flow of cold air, was considered in^10^. Freezing of a drop consists of two main processes: cooling of the drop to the phase transition temperature and subsequent freezing of the spherical layer of the ice shell. At the same time, the shape of a water drop at R < < 2.5 mm^11–14^, i.e. at a small Bond number, it can be considered almost spherical. Water droplets with a size of less than 100 microns should not explode due to the expansion of water upon freezing^15^.

**Figure 1:**
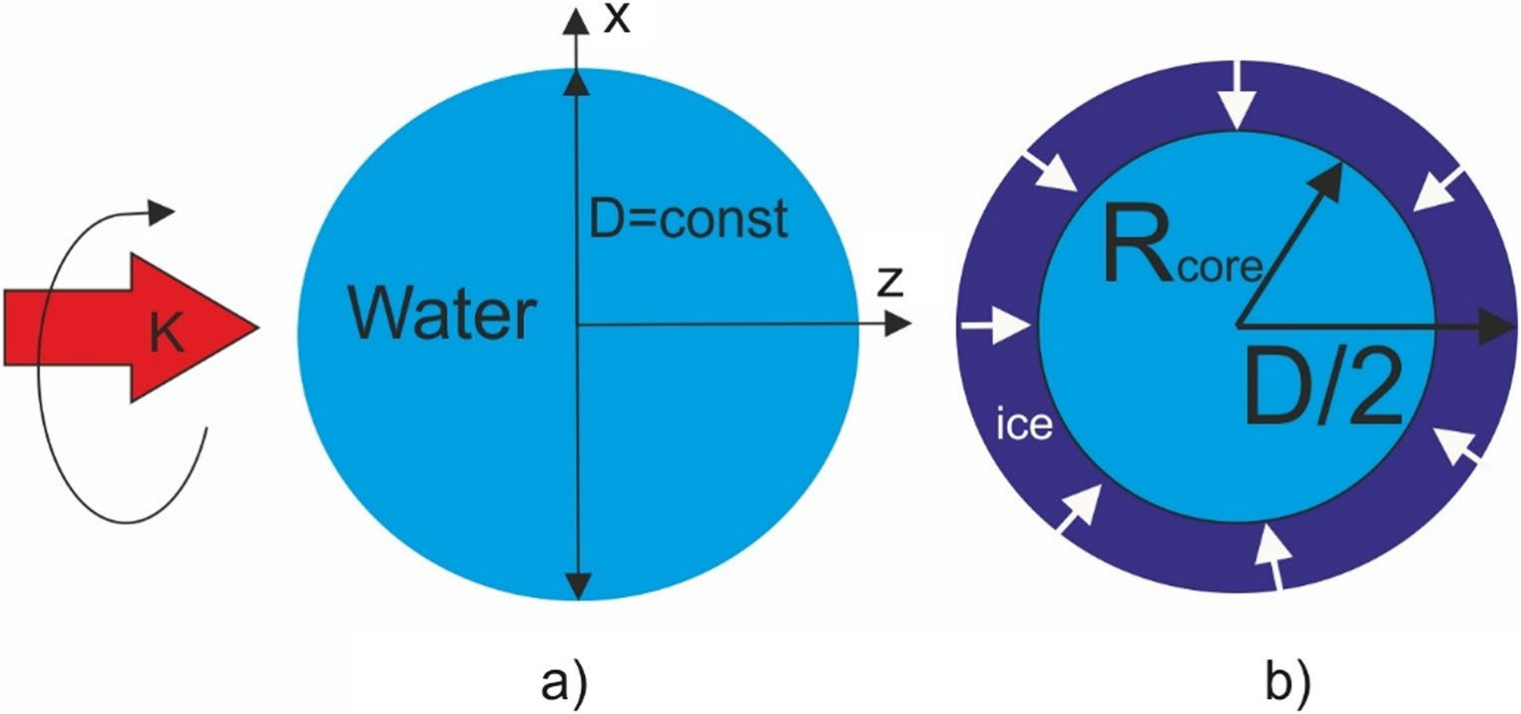
Schematic diagram of the initial stage of the outside in water droplet freezing.

Light scattering in a system of a spherical water particle with an ice shell (see Fig. 1) was studied using commercial software COMSOL Multiphysics. An incident plane wave with circular polarization, with electric field strength |*E*_0_| = 1 V/m propagates along the *z*axis. Thanks to spherical symmetry, the dimension of a 3D problem is reduced to 2D^16^, which simplifies the understanding of the field structure. An axisymmetric two-dimensional problem models a real three-dimensional problem by calculating fields in only one half-plane in cylindrical coordinates^16^. A perfectly matched layer was used as the boundary condition. As in^7,11^, the refractive indices of water and solid ice at a wavelength λ = 589 nm are chosen to be 1.334 and 1.301, respectively. Thus, the core-shell refractive index contrast is close to unity (1.025), and the outer diameter of the sphere (droplet) is constant. Inside and outside the water–ice droplet, the grid size is *λ*/20 and *λ*/10, respectively. A drop with an outer diameter *D*= 6 μm corresponds to the mesoscale^17–19^ size parameter *q* ~ 32 in the Mie theory, immersed in air (*n*= 1). Note that for water droplets with a diameter less than the wavelength, the formation of a photonic jet and Fano resonances does not occur^17,18^. The parameter δ in the formula $R_{core}=(1-\delta)D/2$ characterizes the thickness of the ice spherical shell.

The dynamics of droplet freezing is a multistage process, the details of which are still not completely clear^9,15^. At the same time, calculations^20^show that the characteristics of a photon jet generated by a mesoscale sphere with a rough shell (teeth ~ 200 nm, roughness with a correlation length of 0.08 and an amplitude of 50 nm) are practically no different from the characteristics of a photonic jet in an ideal homogeneous drop. This allows us to ignore the details of the formation of the shell itself and focus on phenomena associated with the Fano resonance in a freezing drop. In this case, we assume the simplest scenario of drop freezing, in which the ice-water interface front moves symmetrically into the liquid. The effects of expansion and contraction of the ice layer in a drop with a low Bond number can be neglected^15^.

## Results

**Figure 2:**
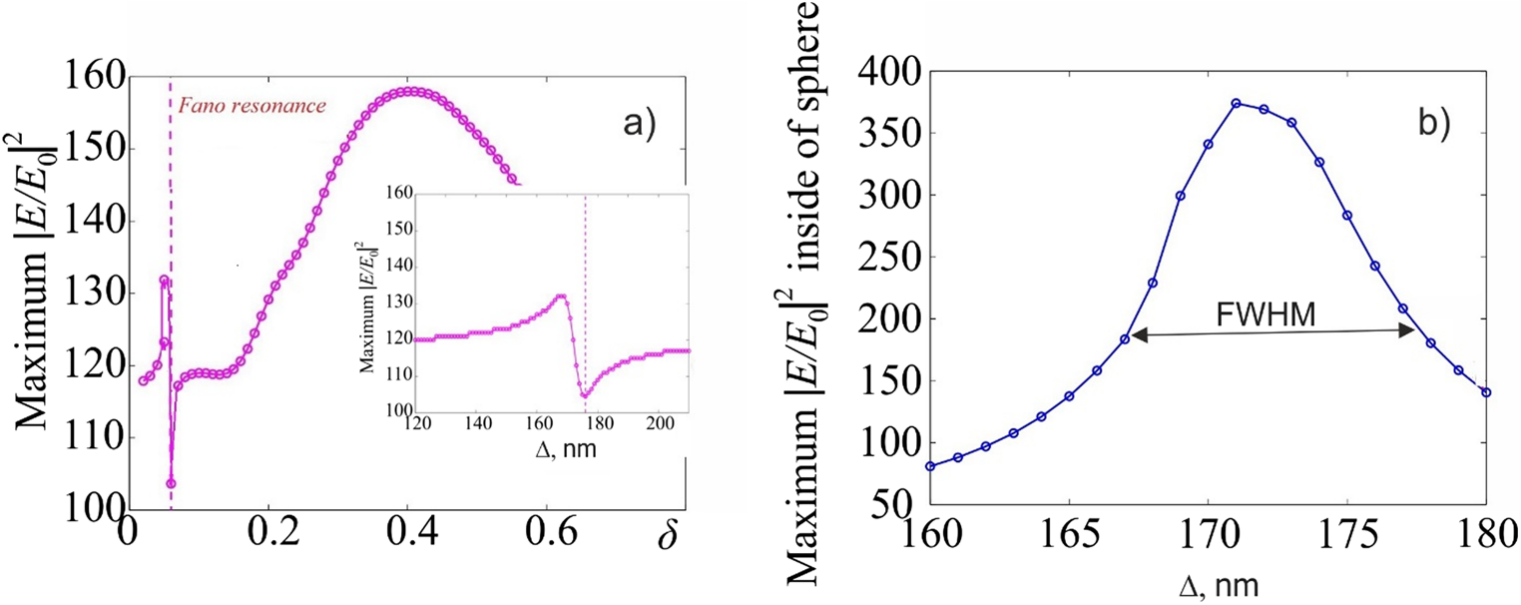
Distribution of maximum relative intensity (E/E 0 ) 2 outside the sphere in the shadow side of the droplet (**a**) vs. δ (**a**). Insert show a zoom image of the resonance position. The dashed line is the position of the minimum electric field intensity along Z-axis inside the droplet. The maximum intensity inside the droplet during Fano resonance near the shadow pole vs. ∆ is shown in (**b**). Here ∆ (in nm) is the dimensional value of the thickness of the ice shell δ.

When a drop freezes, an additional phase shift occurs between its center and periphery, due to the fact that water (core) is optically denser than ice (shell). The temperature (and, accordingly, the refractive index) on both the internal and external surfaces of the shell are determined by the phase equilibria of the ice-vapor and ice-liquid states, respectively^15^. The spherical shape of a drop of the considered size leads to focusing of light^17,18^. The field intensity distribution as a function of the thickness of the ice layer of the droplet are shown in Fig. 2.

Figure 2(b) shows the asymmetric distribution of light intensity along the axis of propagation of the photonic jet. The minimum relative field strength is observed near the value δ ~ 0.05 (Fig. 2a), which corresponds to an ice layer thickness of about 171 nm (see inset in Fig. 2a). This is due to the destructive and constructive interference of scattering light between the shell and the core^21^.

**Figure 3:**
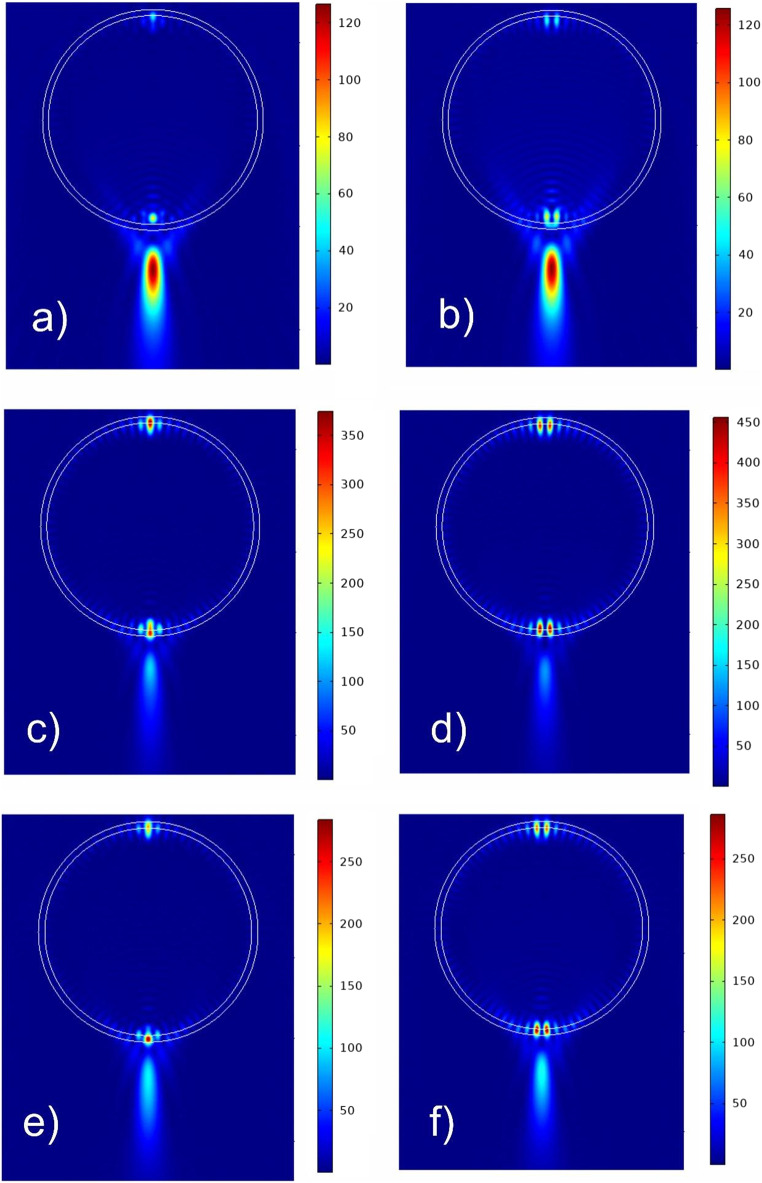
Normalized fields intensity distributions inside and near the shadow surface of the freezing droplet with the thickness of the ice shell: 160 nm (**a**, **b**), 171 nm (**c**, **d**), 175 nm (**d**, **e**) for electric |E/E_0_|^2^ (left column) and magnetic |H/H_0_|^2^ (right column) fields.

Figure 3 shows the normalized distributions of the electric (left column) and magnetic (right column) field strengths inside and near the shadow surface of a freezing drop for characteristic values of the parameter δ. It can be seen that the Fano resonance inside a water drop occurs when the condition 160 nm < *δ* < 180 nm is satisfied (Fig. 2b).

Note that the Mie theory present electric and magnetic fields in the form of multipole expansions, which are presented by some sum, e.g. E = Sum(f_n_), where the total number of terms n_max_is determined by the particle size parameter^18^. Specifics of high order Fano resonance is that the only resonant term *n* = *l* < n_max_ corresponds to maximal value Abs (f_n_ = max) (see e.g. Figure 3in Ref^5^). In our case (Fig. 3c, d) *l* = 76 (Fig. 3c, d).

The field structure inside a freezing drop (Fig. 3c) is typical for the case of a high-order Fano resonance^5,19–21^. The magnetic field strength at resonance exceeds the electric field strength at hot spots at the poles of the drop with *δ* = 171 nm by approximately 1.3 times (Fig. 3c, d). The electric field intensity |E/E_0_|^2^ at resonance is enhanced above 350 times (Fig. 3c), and the magnetic field intensity |H/H_0_|^2^ is enhanced by 450 times (Fig. 3d). Note that far from the Fano resonance, for example at *δ* = 160 nm (Fig. 3a, b), the intensities at the focus of the photonic jet exceed the maximum intensities at hot spots inside the droplet.

According to studies^13,22^, the average speed of propagation of the ice-water boundary in a drop of 10 microns in size freezing water is about 0.028 m/s. Thus, depending on the freezing scenario^13,23,24^, a 10 μm water drop completes freezing in approximately 2.4 ms. According to Fig. 2, the width of the Fano resonance line at half maximum is about 10 nm, so the “lifetime” of the Fano resonance when a water drop of the considered size freezes from outside to inside is about 0.35 µs.

Therefore, below we consider freezing the outside in water droplet with small Bond number in the air at which the shape of the droplet is spherical due to surface tension effect. Time-varying unconventional Fano resonance with magnetic field enhancement yield new method to manipulate light–matter interactions in a freezing water droplet. In principle, a similar effect is possible in other freezing liquids. However, one should keep in mind the specifics of a particular phase transition e.g. due to effects of self-shaping of oil droplets via the formation of intermediate phases upon cooling (see, for example^25^),. This can lead to more complex scattering dynamics. The phase transition can be induced not only by temperature, but also by pressure, as well as by the specific nucleation of the critical nucleus (for example, a disk nucleus instead of a spherical one). At the same time, one should remember that the quality of nonspherical resonator is less than spherical. This also applies to the problem of the freezing droplets on a solid surface^23,24,26^ on which the shape of the droplet al.ways is not a spherical due to the contact angle is less 180 degree. Moreover, the “heterogeneous freezing” on the surface is a separate and complex problem. Thus, we expect less efficient generation of electromagnetic field for nonspherical droplet.

The fact that a change in the thickness of the ice shell in a freezing drop of water leads to significant changes in the intensities of electric and magnetic fields inside the drop, in our opinion, opens up new prospects in various practical areas. For example, it is known that magnetic field oscillations resulting from atmospheric events could have an effect on growth and development of plants and on the responsive reactions of plants to other environmental factors (see e.g^27,28^). There is also magnetobiology, a science that studies the response of living systems to the action of a magnetic field. In recent decades, significant progress has been made in studying the response of animals, as opposed to plants, to the action of a magnetic field. The behavior of a hedgehog in the freezing fog represents a page unknown to science.

## Conclusion

Our motivation for the current research is that despite the large number of publications in which Fano resonance in dielectric core-shell spheres was considered, all of them were devoted to the stationary problem. Moreover, the absolute number of publications is devoted to dielectrics with a high refractive index (more than 2). In this paper, we report the discovery of a Fano resonance in a dielectric core-shell sphere (water-ice) with a low refractive index (1.3) and with a low refractive index contrast between the shell (ice) and core (water) materials. Moreover, the nature of the Fano resonance is essentially non-stationary, since the resonance occurs when a drop of water freezes from the outside in at a certain thickness of the ice shell. Note also that the core-shell sphere is observed in nature during fog and precipitation. These phenomena are new and have not been considered in the literature before.

We show that a change in the thickness of the ice shell in a freezing drop of water leads to significant changes in the intensities of the electric and magnetic fields inside the drop. Using numerical modeling, we studied the fields in a typical mesoscale freezing drop of water, typical for atmospheric physics^29^, and discovered the formation of new optical effects caused by high-order Fano resonance, previously not considered in the literature. This resonance leads to a significant increase in electric and magnetic fields at hot spots at the poles of the drop. The formation of such a resonance allows us to better understand the nature of phenomena in freezing drops and opens up the new prospects in various practical areas.

Of course, experimental studies of the phenomenon under consideration would be very useful. The measurement can be based on the technique described, for example, in Ref^15^. with appropriate modification to ensure the measurement of magnetic and electric fields in the vicinity of the poles of the droplet. However, such measurements are not trivial^30,31^ and will require significant technological efforts in the future.

## Data Availability

The datasets analyzed during the current study available from the corresponding author on reasonable request.
